# Supplementary material for: Intra-Host Evolution During Relapsing Parvovirus B19 Infection in Immunocompromised Patients
Source: Viruses. 2025 Jul 23;17(8):1034. doi: 10.3390/v17081034 (PMC12390661; doi:10.3390/v17081034)
Supplement: Supplementary file 1 [file viruses-17-01034-s001.zip › Russcher_Supplemental_Powerpoint S1.pptx]

## Slide 1
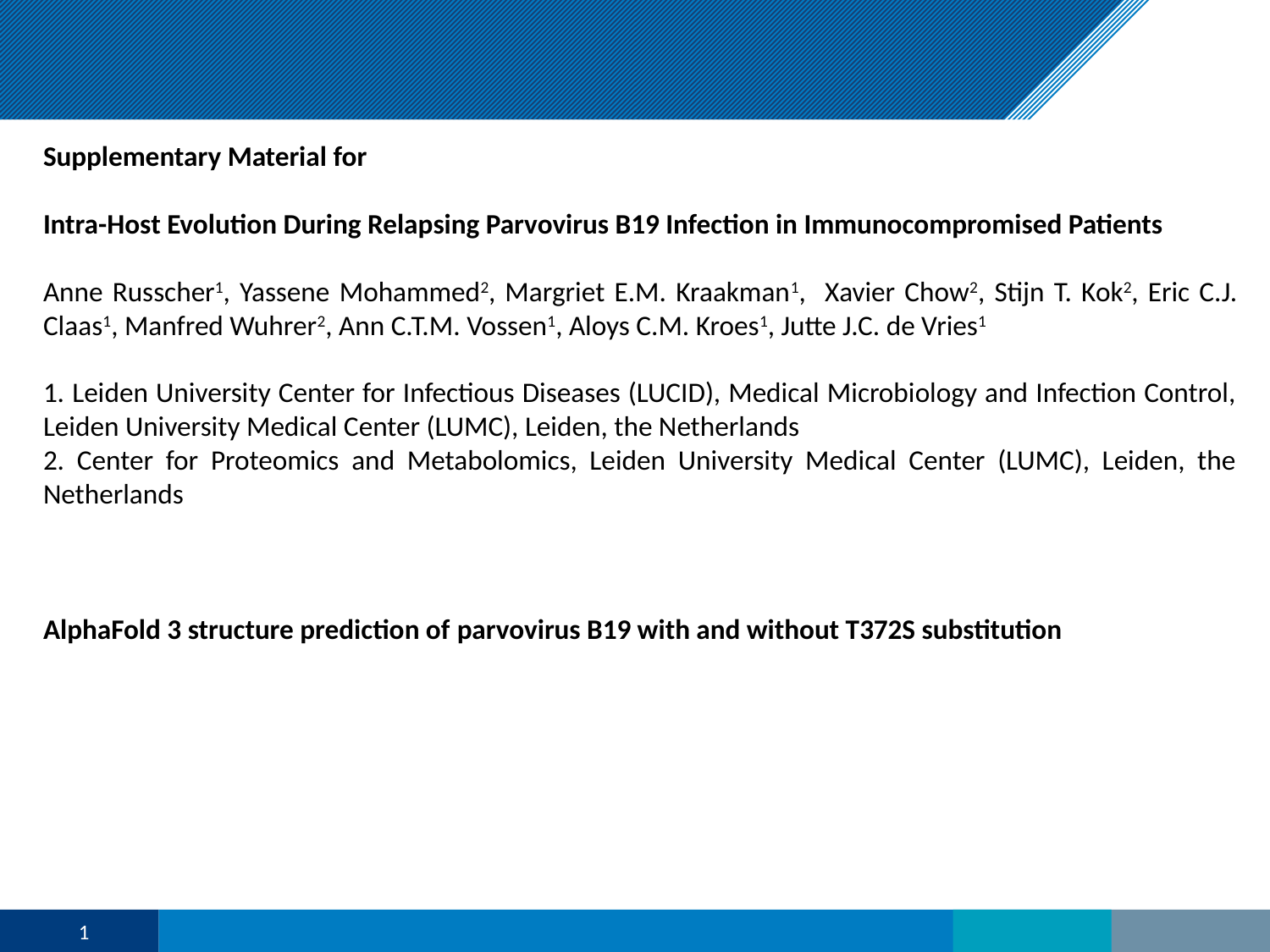

Supplementary Material for
Intra-Host Evolution During Relapsing Parvovirus B19 Infection in Immunocompromised Patients
Anne Russcher1, Yassene Mohammed2, Margriet E.M. Kraakman1, Xavier Chow2, Stijn T. Kok2, Eric C.J. Claas1, Manfred Wuhrer2, Ann C.T.M. Vossen1, Aloys C.M. Kroes1, Jutte J.C. de Vries1
1. Leiden University Center for Infectious Diseases (LUCID), Medical Microbiology and Infection Control, Leiden University Medical Center (LUMC), Leiden, the Netherlands
2. Center for Proteomics and Metabolomics, Leiden University Medical Center (LUMC), Leiden, the Netherlands
AlphaFold 3 structure prediction of parvovirus B19 with and without T372S substitution
1

## Slide 2
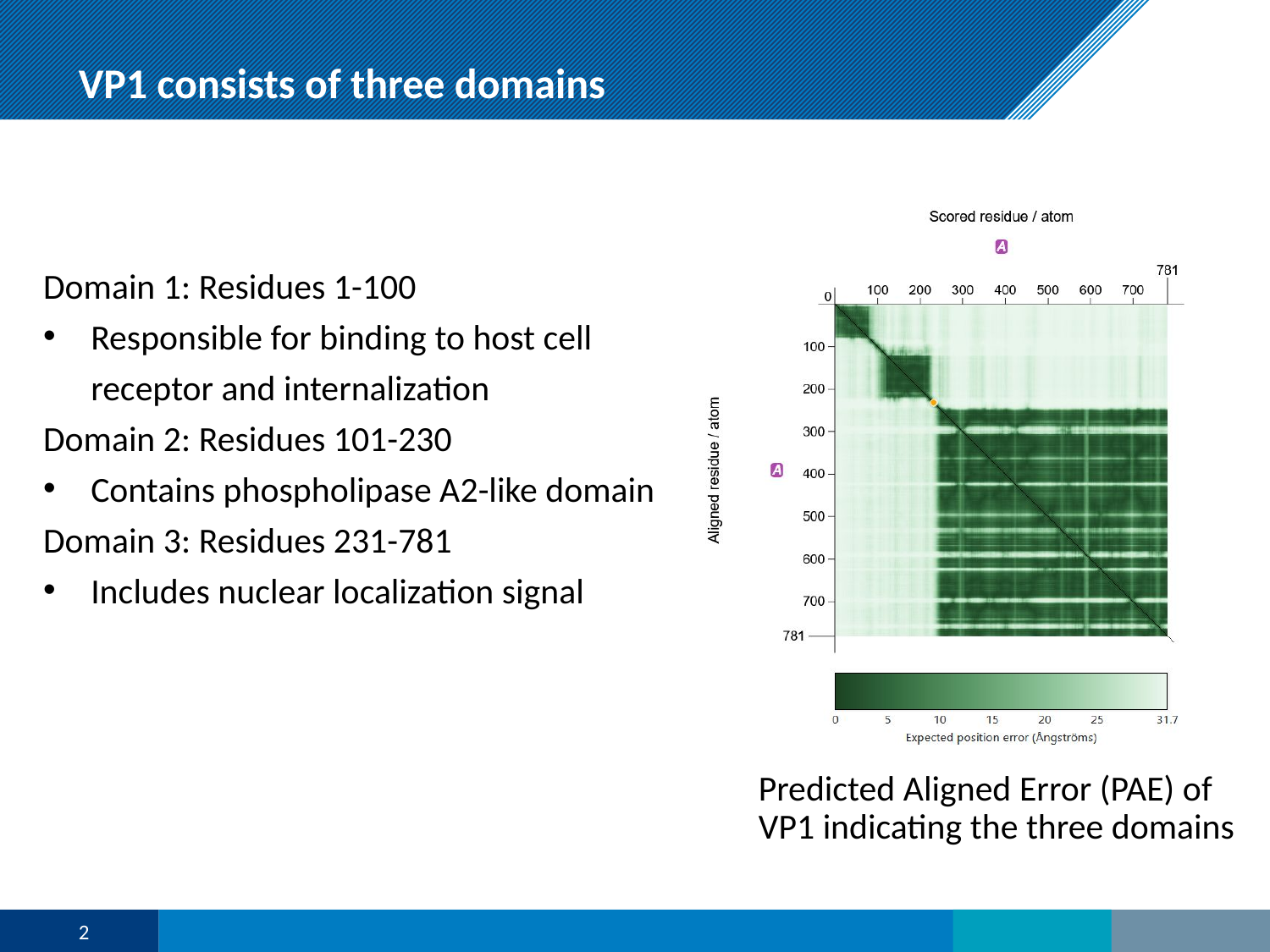

# VP1 consists of three domains
Domain 1: Residues 1-100
Responsible for binding to host cell receptor and internalization
Domain 2: Residues 101-230
Contains phospholipase A2-like domain
Domain 3: Residues 231-781
Includes nuclear localization signal
Predicted Aligned Error (PAE) of VP1 indicating the three domains
2

## Slide 3
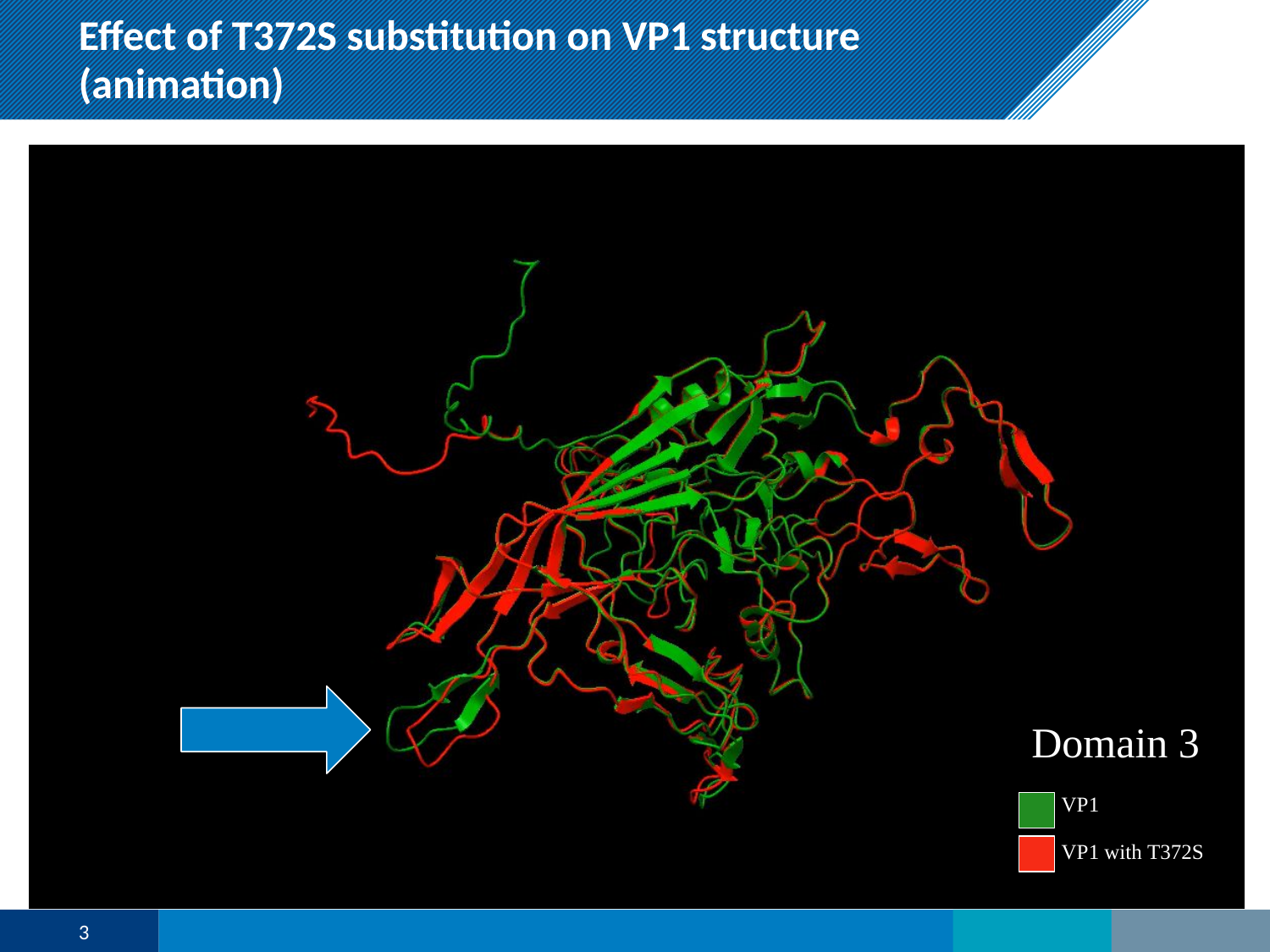

# Effect of T372S substitution on VP1 structure (animation)
Domain 3
VP1
VP1 with T372S
3
