# Supplementary material for: Intra-Host Evolution During Relapsing Parvovirus B19 Infection in Immunocompromised Patients
Source: Viruses. 2025 Jul 23;17(8):1034. doi: 10.3390/v17081034 (PMC12390661; doi:10.3390/v17081034)
Supplement: Supplementary file 1 [file viruses-17-01034-s001.zip › Russcher_Supplemental_Figures S1_S2_Table S1.pdf]

Supplementary Materials

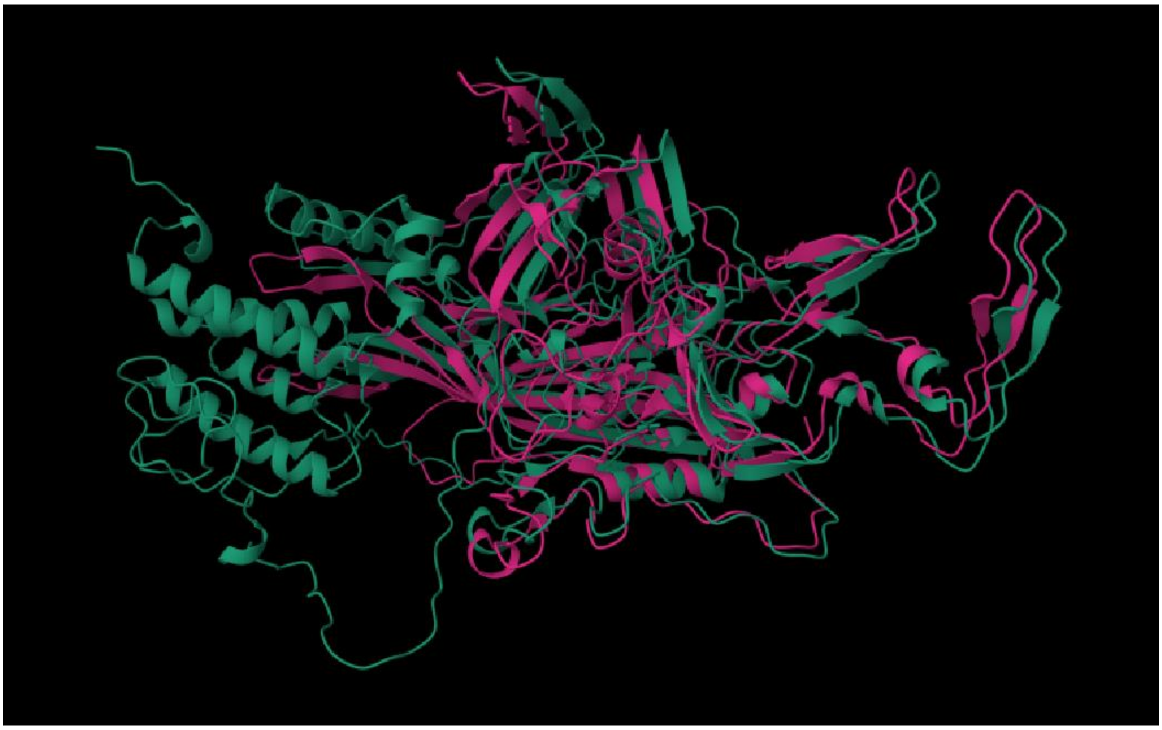

Figure S1. Protein structural deep learning modeling of VP1 (green) and VP2 (pink) in a superimposed view (patient A, time point 1). *The area of VP1 that does not overlap with VP2 is the VP1 unique region, which holds the receptor binding domain.*

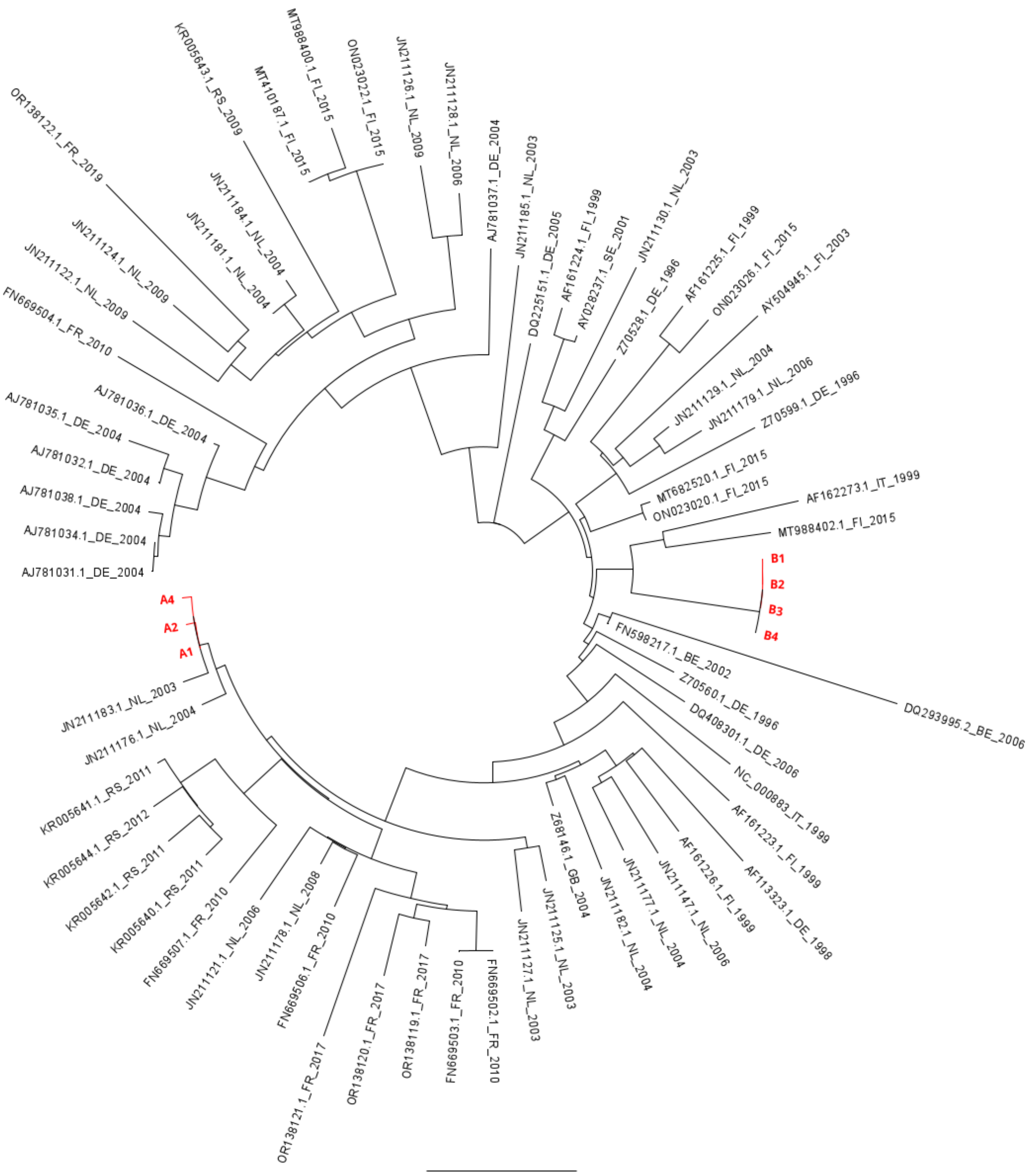

Figure S2: Phylogenetic analysis of NS1, VP1/2 (4280 bp) of study isolates in relation to other European genotype 1 strains*. GenBank strains are denoted by GenBank entries, followed by country (2-digit code according to ISO-3166-1 alpha 2-country codes) and year of isolation.*

Table S1: Literature overview of studies on population evolution of B19V.

First Author	Year	Study Population	Geographical Sampling Location	Sampling Period	Sequencing Method	Results
Gallinella	1995	Blood samples from 7 known B19V patients; nt 2400–3400 (VP) sequenced	Italy	1989–1994	Sanger	No s/s/y* reported; divergence rate within geographic cluster similar to global divergence
Shackelton	2006	Dataset B19V genotype 1: 38 VP1 sequences; 27 NS1–VP1 sequences	Europe (England, Germany, Finland, Ireland, Sweden); USA; Brazil; Japan; Korea; China	1973–2001	Gen Bank sequences	Both VP1 and NS1: ~1 x 10^{-4} nucleotide s/s/y
Norja	2008	63 blood or autopsy samples; Nt 3242–4612 (VP) sequenced	Europe (Germany, Finland, UK)	1980–2007	Sanger	4 x 10^{-4} s/s/y
Suzuki	2008	104 NS1–VP1 junction sequences	Sapporo (Japan)	1980–2008	Sanger	No s/s/y reported; 2 distinct patterns observed in small region: 1. Accumulation of SNP and 2. Sudden replacement of strains
Toppinen	2015	43 bone samples^; part of NS1/VP sequenced	Finland	WWII casualties	Sanger (?)	1.1–3.2 x 10^{-4} s/s/y
Stamenkovic	2016	137 Gen Bank sequences nt 665–4851	Europe; USA; Brazil; Japan; Vietnam	1973–2012	Gen Bank sequences	1.03 x 10^{-4} s/s/y
Mühlemann	2018	10 samples of preserved ancient B19V from teeth (63.9–99.7% coverage)	Eurasia; Southeast Asia; Greenland	~4800 BC–1000 AD	NGS	1.22 x 10^{-5} s/s/year (strict clock)
Guzman-Solís	2021	3 samples of preserved ancient B19V from teeth (92.4–99.1% coverage)	Mexico	16th–18th century	NGS	1.03 x 10^{-5} s/s/y (strict clock)

* s/s/y = substitutions/site/year; ^ bone material from World War II (WWII) casualty graves.

References table S1

1. Gallinella, G.; Venturoli, S.; Gentilomi, G.; Musiani, M.; Zerbini, M. Extent of sequence variability in a genomic region coding for capsid proteins of B19 parvovirus. *Arch. Virol.* **1995**, *40*, 1119–1125. https://doi.org/10.1007/BF01315420.
2. Shackelton, L.A.; Holmes, E.C. Phylogenetic evidence for the rapid evolution of human B19 erythrovirus. *J. Virol.* **2006**, *80*, 3666–3669. https://doi.org/10.1128/JVI.80.7.3666-3669.2006.
3. Norja, P.; Hokynar, K.; Aaltonen, L.-M.; Chen, R.; Ranki, A.; Partio, E.K.; Kiviluoto, O.; Davidkin, I.; Leivo, T.; Eis-Hübinger, A.M.; et al. Bioportfolio: Lifelong persistence of variant and prototypic erythrovirus DNA genomes in human tissue. *Proc. Natl. Acad. Sci. USA* **2006**, *103*, 7450–7453. https://doi.org/10.1073/pnas.0602259103.
4. Suzuki, M.; Yoto, Y.; Ishikawa, A.; Tsutsumi, H. Analysis of nucleotide sequences of human parvovirus B19 genome reveals two different modes of evolution, a gradual alteration and a sudden replacement: A retrospective study in Sapporo, Japan, from 1980 to 2008. *J. Virol.* **2009**, *83*, 10975–10980. https://doi.org/10.1128/JVI.00273-09.
5. Toppinen, M.; Perdomo, M.F.; Palo, J.U.; Simmonds, P.; Lycett, S.J.; Soderlund-Venermo, M.; Sajantila, A.; Hedman, K. Bones hold the key to DNA virus history and epidemiology. *Sci. Rep.* **2015**, *5*, 17226. https://doi.org/10.1038/srep17226.
6. Stamenković, G.G.; Ćirković, V.S.; Šiljić, M.M.; Blagojević, J.V.; Knežević, A.M.; Joksić, I.D.; Stanojević, M.P. Substitution rate and natural selection in parvovirus B19. *Sci. Rep.* **2016**, *6*, 35759. https://doi.org/10.1038/srep35759.
7. Mühlemann, B.; Margaryan, A.; Damgaard, P.d.B.; Allentoft, M.E.; Vinner, L.; Hansen, A.J.; Weber, A.; Bazaliiskii, V.I.; Molak, M.; Arneborg, J.; et al. Ancient human parvovirus B19 in Eurasia reveals its long-term association with humans. *Proc. Natl. Acad. Sci. USA* **2018**, *115*, 7557–7562. https://doi.org/10.1073/pnas.1804921115.
8. A Guzmán-Solís, A.; Villa-Islas, V.; Bravo-López, M.J.; Sandoval-Velasco, M.; Wesp, J.K.; A Gómez-Valdés, J.; Moreno-Cabrera, M.d.l.L.; Meraz, A.; Solís-Pichardo, G.; Schaaf, P.; et al. Ancient viral genomes reveal introduction of human pathogenic viruses into Mexico during the transatlantic slave trade. *Elife* **2021**, *10*, e68612. https://doi.org/10.7554/eLife.68612
